# Plecoptera of Canada

**DOI:** 10.3897/zookeys.819.23535

**Published:** 2019-01-24

**Authors:** Boris C. Kondratieff, R. Edward DeWalt, Chris J. Verdone

**Affiliations:** 1 Colorado State University, Department of Bioagricultural Sciences and Pest Management, 1177 Campus Delivery, Fort Collins, Colorado 80523, USA Colorado State University Fort Collins United States of America; 2 University of Illinois, Prairie Research Institute, Illinois Natural History Survey, 1816 S Oak Str., Champaign, Illinois 61820, USA University of Illinois Champaign United States of America

**Keywords:** biodiversity assessment, Biota of Canada, Plecoptera, stoneflies

## Abstract

Currently, a total of 267 stonefly species are known for Canada. The biodiversity hotspot of Canadian stoneflies is British Columbia with at least 138 species, nearly 52% of all species known from Canada. Four families, the Perlodidae, Capniidae, Chloroperlidae, and Nemouridae, contain nearly 75% of all species known to occur in Canada. The family with the fewest species represented in Canada is the Peltoperlidae. The stonefly fauna of Canada consists of two major faunal assemblages, west and east. The western clade consists of those species inhabiting Manitoba, all provinces to the west, and the three territories. The eastern clade consists of species from Ontario eastward. The two clades share only 29 species (10.9% of the Canadian total), suggesting a separate origin for each clade. The available taxonomic literature for the stoneflies of Canada is reviewed.

The order Plecoptera, or stoneflies, a small group of hemimetabolous insects, includes approximately 3700 extant, valid species placed in 16 families worldwide (Fochetti and Tierno de Figueroa 2008, DeWalt et al. 2018). Diversity is often highest in temperate montane regions of the world (Fochetti and Tierno de Figueroa 2008). More than for any other order of insects, larval stoneflies are typical inhabitants of lotic habitats (Hynes 1976); larvae of some species are also known to inhabit cold, oligotrophic lakes at high latitudes and altitudes, such as in the Canadian boreal and alpine areas (Harper 1979, Donald and Anderson 1980, Dosdall and Lehmkuhl 1987, Stewart and Oswood 2006). Most North American stonefly families have also radiated into warmer streams as a result of the evolution of physiological traits such as embryonic or larval diapause (Stewart and Stark 2002). This adaptation to a wide range of conditions makes stoneflies useful for monitoring water quality (Baumann 1979).

All Canadian stoneflies belong to one of the two suborders, the Arctoperlaria, which is further separated into two “groups” (DeWalt et al. (2018) consider them infraorders), the Systellognatha and Euholognatha (Zwick 2000). Larvae of the former are generally predaceous on other aquatic invertebrates, while the latter are detritivores, eating dead leaves and wood conditioned by microbes (Stewart and Stark 2002).

The stoneflies of North America, north of Mexico, are relatively well-known with at least 778 extant, valid species and subspecies (DeWalt et al. 2018). The earliest mention of a species of stonefly occurring in Canada was that of the Holarctic *Diurabicaudata* (Linnaeus, 1758). Ricker (1964) provided the first comprehensive review of the stoneflies of Canada, including a historical overview of Canadian stoneflies. Ricker (1964) indicated that about 202 species were known from Canada at that time, and estimated that perhaps 12 additional species had been collected but were yet undescribed.

Fourteen years later, Harper (1979) indicated that 250 species were known from Canada, and estimated that another 60 species were likely present but were yet undescribed or undiscovered. He indicated that 124 species were known from eastern Canada and 135 from the west, the overlap of taxa in central Canada being only nine species. Unfortunately, he did not state how he delineated the eastern and western regions of Canada.

Stonefly species lists or records are available for all ten Canadian provinces and three territories: Alberta (Ricker 1946, Donald and Anderson 1977, Stewart and Oswood 2006, Dosdall and Giberson 2014a, b), British Columbia (Banks 1907, Ricker 1939, 1943, Ricker and Scudder 1975, Scudder 1994, Stewart and Oswood 2006, Baumann and Stark 2010, Dosdall and Giberson 2014a, b), Manitoba (Ricker 1946, Flannagan 1978, Burton 1984, Flannagan and Cobb 1983, Dosdall and Giberson 2014a, b), New Brunswick (Ricker 1948, Kondratieff and Baumann 1994, Giberson and Garnett 1996), Newfoundland and Labrador (Banks 1908, Ricker 1944, 1948, Brinck 1958), Northwest Territories (Ricker and Judd 1955, Stewart and Oswood 2006, Vinke et al. 2015), Nova Scotia (Ricker 1948, Kondratieff and Baumann 1994), Nunavut (Stewart and Oswood 2006), Prince Edward Island (Kondratieff and Baumann 1994, Dobrin and Giberson 2003), Ontario (Harper and Ricker 1994), Québec (Ricker et al. 1968, Harper et al. 1975, 1991a, b, Harper 1990), Saskatchewan (Ricker 1946, Dosdall 1976, Dosdall and Lehmkuhl 1979, 1987, Miyazaki and Lehmkuhl 2011, Dosdall and Giberson 2014a, b), and the Yukon (Stewart and Ricker 1997, Stewart and Oswood 2006).

Additional useful treatments of regional stonefly faunas that include Canadian species are Hitchcock (1974) for eastern Canada, Jewett (1959) and Baumann et al. (1977) for British Columbia and Alberta, and Szczytko and Stewart (1979) for western North American *Isoperla* Banks. Recently, Szczytko and Kondratieff (2015a, b) revised the eastern North American species of the Isoperlinae based on adults, recording 20 species from Canada. Baumann and Stark (2013) revised the genus *Megaleuctra* Neave and provided records for Alberta, British Columbia, and possibly Manitoba. Stewart and Oswood (2006) provided much information about the stoneflies of western Canada. Additionally, Dosdall and Giberson (2014a, b) provided a useful synopsis of the stoneflies of Alberta, Saskatchewan, and Manitoba. Danks (1981) presented a list of known Canadian Arctic plecopteran species.

Reliable keys exist for identification of both adult and larval stoneflies. Larvae can be identified to genus using Stewart and Stark (2002, 2008) and DeWalt and Kondratieff (in press); most adults to species using Stark and Armitage (2000, 2004) and Stewart and Oswood (2006). Many of the classic publications in North American plecopterology (e.g., Needham and Claassen 1925, Frison 1942, Ricker 1952, Ross and Ricker 1971) are also useful for studying the Canadian fauna. Harper and Hynes (1971a–d) provide keys to adults and immatures of euholognathan species of eastern Canada.

Despite the stoneflies of Canada being relatively well documented, additional collecting is required to fully understand the fauna. For example, *Isoperlacitronella* (Newport) described from St. Martins Falls, Ontario is still apparently known from only two female specimens (Harper and Ricker 1994, Szczytko and Kondratieff 2015a), and no adult male has been positively associated. Canadian regions that need additional surveys include the Prairie Provinces, coastal British Columbia, western and northern Ontario, and the more remote areas of the Yukon and Northwest Territories. Particular attention in the eastern provinces should be paid to study of the small, summer emerging perlids *Neoperla* Needham and *Perlesta* Banks, both of which are surely represented by more species than are currently known.

A general discussion of stonefly biology and ecology is presented in DeWalt et al. (2015), while a synopsis of the ecological information for North American stoneflies, including Canadian species, is presented in Stewart and Stark (2002). Specific ecological information is available for about 50 Canadian species. For example, life histories for many species have been reported by Harper and Magnin (1969), Coleman and Hynes (1970), Harper and Pilon (1970), Harper and Hynes (1972), Harper (1973a, b), Barton (1980), Mutch and Pritchard (1982, 1984), Harper et al. (1991a, b), and Dobrin and Giberson (2003).

A presence/absence species-by-province data matrix was created using data stored in Plecoptera Species File (PSF), a web-based, global resource for information about stoneflies (DeWalt et al. 2018). The list of species known from each province is available as Suppl. material 1 in Comma Separated Values format. The relationship of province assemblages to each other was analyzed using the R package *vegan*. A Jaccard distance matrix for pairwise distances between samples was constructed using the *vegdist* function. This matrix was used to perform an agglomerative cluster analysis based on Jaccard average linkage with the function *hclust*. The province relationships were displayed as a dendrogram.

Currently, a total of 267 stonefly species have been recorded from Canada (Table 1). Four families, Perlodidae, Capniidae, Chloroperlidae, and Nemouridae contain nearly 75% of all species known to occur in Canada (Fig. 1). The family with the fewest species represented in Canada is the Peltoperlidae.

**Table 1.** Census of Plecoptera in Canada.

**Taxon^1^**	**No. species reported in Harper (1979)**	**No. species currently known from Canada**	**No. BINs^2^ available for Canadian species**	**Est. no. undescribed or unrecorded species in Canada**	**General distribution by ecozone^3^**	**Information sources**
**Order Plecoptera**						Baumann et al. 1977, Stewart and Stark 2002, Stewart and Oswood 2006, DeWalt et al. 2018, DeWalt and Kondratieff (in press)
**Suborder Arctoperlaria**						
**Infraorder Euholognatha**						
Capniidae	49	52	19	2	all ecozones	
Leuctridae	21	22	20	2	all but Arctic	
Nemouridae	32	41	45	5	all ecozones	
Taeniopterygidae	15	14	9	0	all but Arctic	Stewart 2000
**Infraorder Systellognatha**						
Peltoperlidae	4	5	1	0	Pacific Maritime, Montane Cordillera, Boreal Shield	Stewart 2000
Pteronarcyidae	9	9	2	0	all ecozones	Nelson 2004, Myers and Kondratieff 2017
Chloroperlidae	44	46	33	5	all but Arctic	Surdick 2004
Perlidae	15	18	13	10	all but Arctic	Stark 2004
Perlodidae	61	60	24	10	all ecozones	Kondratieff 2004, Szczytko and Kondratieff 2015a, b
**Total**	**250**	**267**	**166**	**34**		

**^1^**Classification from DeWalt et al. (2018). **^2^**Barcode Index Numbers as defined by Ratnasingham and Hebert (2013). **^3^** See figure 1 in Langor (2019) for a map of ecozones.

**Figure 1.**
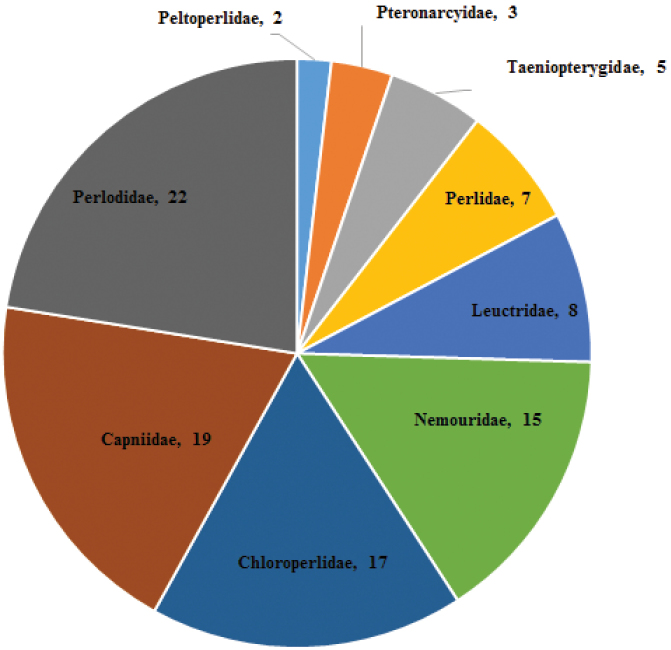
The percent of the Canadian Plecoptera fauna represented by each family. The numbers represent rounded percentages and do not total 100.

Efforts have been made to barcode North American species of stoneflies including Canadian taxa (i.e., Ratnasingham and Hebert 2013, Zhou et al. 2009, Cordero et al. 2017). To date, 166 Barcode Index Numbers (BINs) from Canadian specimens are included in the Barcode of Life Data (BOLD; Ratnasingham and Hebert 2013) database, suggesting that 62% of the recorded number of species found in Canada is represented in their sequence library (Table 1). The Perlidae and Chloroperlidae appear to be well represented with BINs accounting for up to 72% of the recognized fauna. Alternatively, the Capniidae and Perlodidae are under-represented with only 36% and 40% representation, respectively. The number of BINs indicates that there are potentially five additional species of Nemouridae in Canada than are currently recognized (Table 1).

The biodiversity hotspot of Canadian stoneflies is British Columbia with at least 138 species, nearly 52% of all species known from Canada (Fig. 2). This is due to the great density of high gradient streams that dominate the province. Alberta supports the second highest number of species in Canada, and much of its diversity is contained within the eastern extension of the Montane Cordillera ecozone. Other provinces in the west hold fewer species. The Yukon Territory, dominated by Boreal and Taiga Cordillera ecozones with many swiftly flowing streams, support only 71 species. It is probable that this territory holds many more species yet undiscovered due to difficult access to much of the land-base. Northwest Territories and Nunavut are both dominated by Taiga Plains, Taiga Shield, and Arctic ecozones. Streams in these areas are lower gradient and/or frozen for all but a few months of the year, conditions not conducive to a rich stonefly fauna. Difficult access to these territories limits complete understanding of the faunal composition. Reported stonefly diversity in the prairie provinces of Saskatchewan and Manitoba is apparently low, but greater than that of the two territories to their north.

**Figure 2.**
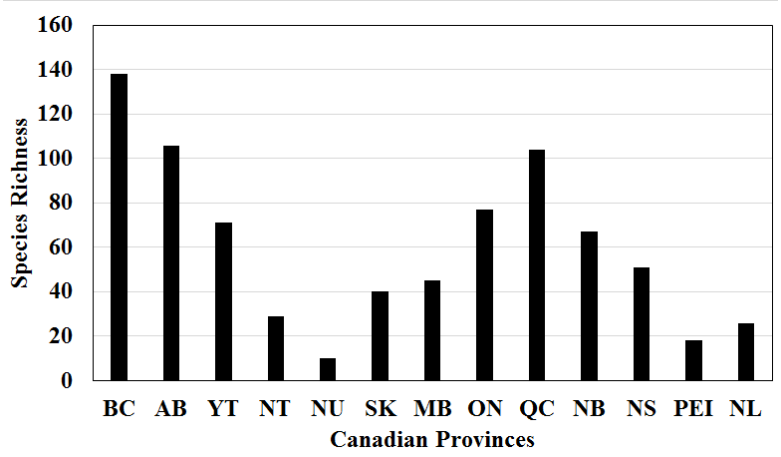
Plecoptera species richness by Canadian provinces. Abbreviations: AB – Alberta, BC – British Columbia, MB – Manitoba, NB – New Brunswick, NL – Newfoundland and Labrador, NS – Nova Scotia, NT – Northwest Territories, NU – Nunavut, ON – Ontario, PEI – Prince Edward Island, QC – Quebec, SK – Saskatchewan, and YT – Yukon.

The province of Quebec (104 species, 39.0% of the national fauna) has the highest species richness of the eastern provinces. The presence of the northern extensions of the Appalachian Mountains and rugged Boreal Shield topography has resulted in a relative diverse assemblage of stoneflies. Ontario is also relatively rich by eastern standards. Despite being well collected, there has not been a detailed treatment of the Ontario fauna since the late 1960s. Many revisions and descriptions of new species have occurred since then that could increase the number of species known in the province by 10–15% (R DeWalt unpubl. data). Further eastward, the Atlantic provinces of New Brunswick and Nova Scotia support a substantial subset of the Quebec assemblage. Conversely, the province of Newfoundland and Labrador supports a much smaller number of stoneflies, perhaps owing to the regional composition of Taiga Shield and Arctic ecozones. Prince Edward Island, with a total area of only 5600 km^2^, has had most of its larger streams impacted by sedimentation due to agricultural activities (Eedy and Giberson 2007). Higher stonefly diversity does occur in cold spring-brooks (Dobrin and Giberson 2003), but represents a small fraction of eastern Canadian stonefly species. Isolation from mainland colonization sources also limits the stonefly fauna of this island.

The Canadian Plecoptera consists of two distinct faunal assemblages, west and east (Fig. 3). Here we define the western fauna as those species inhabiting Manitoba, all provinces to the west, and the three territories. The eastern fauna consists of species from Ontario eastward. These clades are distinctive, sharing only 29 species (10.9% of the Canadian total), 21 species with eastern affinity, suggesting separate origins for each clade. This break in eastern and western clades was also found, though less distinctly, by Nelson (2008).

**Figure 3.**
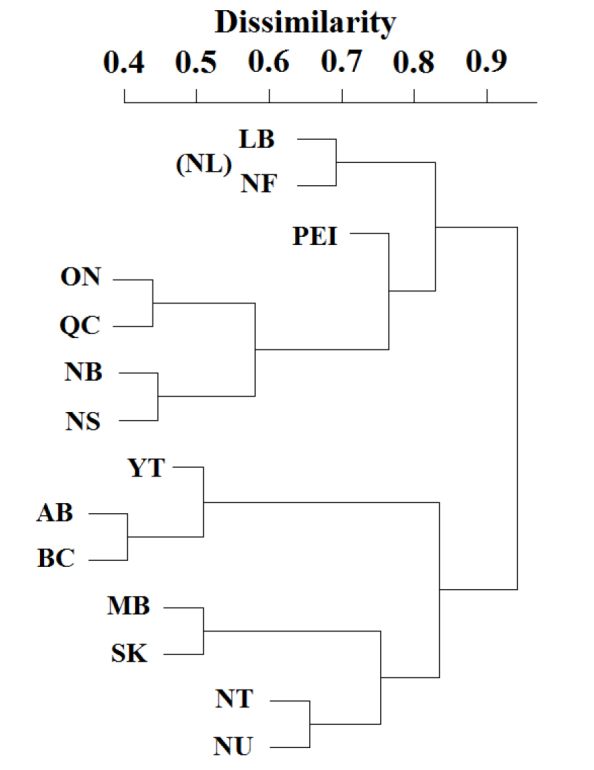
Cluster analysis of Plecoptera assemblages for Canadian provinces. For meaning of province abbreviations, see Figure 2 legend. The assemblages of Newfoundland (NF) and Labrador (LB) were kept separate in this analysis.

## Supplementary materials

Supplementary material 1 List of species known from each province Link: https://doi.org/10.3897/zookeys.819.23535.suppl1 Data type: species data This is an open access article distributed under the terms of the Creative Commons Attribution License (CC BY 4.0), which permits unrestricted use, distribution, and reproduction in any medium, provided the original author and source are credited.Boris C. Kondratieff, R. Edward DeWalt, Chris J. Verdone
